# Supplementary material for: Excessive C5 conversion prevents C9 polymerisation and subsequent MAC-dependent killing of Klebsiella pneumoniae
Source: PLoS Pathog. 2026 May 11;22(5):e1013818. doi: 10.1371/journal.ppat.1013818 (PMC13193605; doi:10.1371/journal.ppat.1013818)
Supplement: S1 Table — (DOCX) [file ppat.1013818.s005.docx]

| Clinical isolate name | KpO2_2 | KpO1_1 | KpO2_1 | Kp209_CSTR | KpO5_2 | KpO3_4 | Kp209 | KpO5_1 | KpO3_3 |
| --- | --- | --- | --- | --- | --- | --- | --- | --- | --- |
| ST | ST307 | ST219 | ST219 | ST11 | ST187 | ST37 | ST11 | ST17 | ST2670 |
| wzi | wzi173 | wzi177 | wzi177 | - | wzi10 | wzi96 | - | wzi141 | wzi193 |
| K_locus | KL102 | KL114 | KL114 | KL110 | KL10 | KL38 | KL110 | KL25 | KL125 |
| K_type | unknown (KL102) | unknown (KL114) | unknown (KL114) | unknown (KL110) | K10 | K38 | unknown (KL110) | K25 | unknown (KL125) |
| K_locus_identity | 99.66% | 99.85% | 99.85% | 99.75% | 98.71% | 99.79% | 99.75% | 98.92% | 93.60% |
| O_locus | OL2α.2 | OL2α.1 | OL2α.1 | OL2α.1 | OL5 | OL3γ | OL2α.1 | OL5 | OL3γ |
| O_type | O2β | O1αβ,2α | O2α | O2α | O5 | O3γ | O2α | O5 | O3γ |
| O_locus_identity | 99.13% | 99.32% | 99.32% | 99.33% | 98.39% | 99.67% | 99.33% | 98.23% | 99.78% |
| Aminoglycoside genes | aac(3)-IIa.v1^; strB.v1; strA.v1^; aac(6')-Ib-cr.v2 | strB.v1*; aph3-Ia.v1^; strA.v1*; aadA2^ | strB.v1*; aph3Ia.v1^; strA.v1*; aadA2^ | - | - | strB.v1; strA.v1^ | - | strB.v1*; strA.v1* | aac(3)-IIa.v1^; strB.v1; strA.v1^; aac(6')-Ib-cr.v2 |
| Fluoroquinolone genes | qnrB1.v2^; aac(6')-Ib-cr.v2 | qnrS1 | qnrS1 | - | - | qnrB1.v2^ | - | qnrB4 | qnrB1.v2^; aac(6')-Ib-cr.v2 |
| Sulfonamide genes | sul2 | sul1; sul2 | sul1; sul2 | - | sul1 | sul2^ | - | sul1; sul2 | sul2 |
| Tetracycline genes | - | tet(A).v1 | tet(A).v1 | - | tet(D) | tet(A).v1 | - | tet(A).v1 | tet(A).v1 |
| Trimethoprim genes | dfrA14.v2* | dfrA12 | dfrA12 | - | dfrA5 | dfrA14.v2* | - | dfrA14.v2* | dfrA14.v2* |
| Phenicol genes | - | - | - | - | - | - | - | - | - |
| MLS genes | - | Mrx;mphA | Mrx;mphA | - | - | Mrx;mphA | - | Mrx;mphA* | - |
| β-lactamases | OXA-1 | - | - | - | - | - | - | DHA-1^ | OXA-1 |
| ESBLs | CTX-M-15 | CTX-M-15 | CTX-M-15 | - | CTX-M-15^ | CTX-M-15 | - | - | CTX-M-15 |
| Carbapenemases | - | - | - | - | - | - | - | - | - |
| Inhibitor-resistant β -lactamases | - | - | - | - | - | - | - | - | - |
| Colistin genes | - | - | - | - | - | - | - | - | - |
| Tigecycline determinants | - | - | - | - | - | - | - | - | - |
| Hypermucosivity genes | - | - | - | - | - | - | - | - | - |
| String test | - | - | - | - | - | - | - | - | - |
