# Supplementary material for: Excessive C5 conversion prevents C9 polymerisation and subsequent MAC-dependent killing of Klebsiella pneumoniae
Source: PLoS Pathog. 2026 May 11;22(5):e1013818. doi: 10.1371/journal.ppat.1013818 (PMC13193605; doi:10.1371/journal.ppat.1013818)
Supplement: S2 Table — (DOCX) [file ppat.1013818.s006.docx]

| Neonate | Gestational age (weeks+days) | Gestational status  (Preterm is <37 weeks (45)) |
| --- | --- | --- |
| 1 | 29+3 | Preterm |
| 2 | 29+4 | Preterm |
| 3 | 30+0 | Preterm |
| 4 | 32+6 | Preterm |
| 5 | 35+5 | Preterm |
| 6 | 36+3 | Preterm |
| 7 | 37+4 | Term |
| 8 | 39+2 | Term |
