# Supplementary material for: Excessive C5 conversion prevents C9 polymerisation and subsequent MAC-dependent killing of Klebsiella pneumoniae
Source: PLoS Pathog. 2026 May 11;22(5):e1013818. doi: 10.1371/journal.ppat.1013818 (PMC13193605; doi:10.1371/journal.ppat.1013818)
Supplement: Attachment — Submitted filename: Response to Reviewers.docx [file ppat.1013818.s008.docx]

Dear Editor,

We thank the referees for their interest in our study (*Excessive C5 conversion prevents C9 polymerisation and subsequent MAC-dependent killing of Klebsiella pneumoniae*). We are very pleased that the referees consider our findings crucial to the field and well-designed. Based on the comments given by the referees, we have revised our manuscript in the following manner:

1. We have provided novel experimental data to answer additional questions the reviewers proposed.
   a. S2a: Bacterial killing assay at higher serum concentrations.
   b. S1b-h and S2b-c: In time IM-permeabilisation as a read-out for killing over time including shorter timepoints.
2. We have applied minor changes to figures and figure legends (Fig. 1b) for more transparent representation on the number of samples analysed and clearer interpretation of the data (Figure 2c; Figure 4d; line 280) and discussion points (line 435).
3. We now provide more information on the *K. pneumoniae* strains (Table 1 and S1) and the rationale behind selecting these strains (line 146).

In the “point-to-point” response letter below, we provide responses to all the comments raised by the reviewers and explain how we have revised our paper accordingly in detail. All textual changes into the revised manuscript are also highlighted in blue.

We hope that we have addressed all comments with this rebuttal and look forward to your response.

Best regards,

Dr. Bart Bardoel

# Response to reviewers

## Part I - Major revisions

### Reviewer #1

4. Could the authors provide additional details on how the KP strains were selected? KP have significant genetic heterogeneity based on capsule composition that may influence MAC killing so additional details would be helpful re: generalizability. The authors have provided K type and O antigen type, which is helpful, but additional information that may also help the field would be sequence type, antibiotic resistance profiles (if these are clinical or multi-drug resistant), additional details on unpublished strains, clinical isolate versus laboratory strain, etc.

We have clarified in the manuscript that all *K. pneumoniae* (Kpn) strains used in this study are clinical isolates (line 146). These isolates were genotyped by whole‑genome sequencing and analysed using the Kleborate online tool. From this dataset, eight KP clinical isolates were selected to represent a diverse range of O‑ and K‑antigen types. This information has also been added to the revised text (line 146) and we have updated the supplementary table (Table S1.) to include sequence type, antibiotic resistance profiles and hypermucoviscosity genes (line 146) as additional information.

5. The strain nomenclature is challenging to follow - for example, what does Kp01_1 vs. Kp01_2 mean? Recommend simplifying the nomenclature or perhaps grouping by biology (e.g. by O antigen type). Also, Table S1 should probably be included in the main text.

We have clarified the nomenclature in the figure legend of Table 1 (line 179) and have placed the initial Table S1 now as Table 1 in the main text and have adjusted it accordingly. In brief, the nomenclature is from previous publications (doi:10.1038/s41598-024-71487-z; https://doi.org/10.1128/aac.01958-20) for consistency between papers. All strains are named according to its O‑antigen type, with exceptions of Kp209 and Kp209_CSTR. For example, KpO1_1 refers to a Kpn strain expressing the O1 antigen, with ‘_1’ indicating one of the multiple O1-antigen strains in the study.

6. I have used 5% NHS in complement binding assays but not for killing assays - it appears the serum was allowed to kill overnight? If I understand their assay correctly, I would be interested to know if similar effects could be seen at shorter timepoints (e.g. 30 minutes) and higher serum concentrations that maybe more “physiologic.” I think higher concentrations are particularly important to test. If the authors are willing, I would perhaps target their efforts to only a key experiment or two?

All bacterial killing experiments were performed in serum for 90–120 minutes after which the serum reaction is stopped by dilution in PBS. Overnight incubation on LB agar plates is used to detect CFU/ml. To avoid confusion on the interpretation of the assay set-up, we have adapted the explanation of the method section accordingly (line 513-516).

As for analysis at earlier timepoints, our IM-permeabilisation data shown in Fig. 1a provides a dynamic read-out for killing over time, indicating that the difference we observe is consistent over time. We now include the IM-permeabilisation data over time for every strain at 5% NHS (S1b-h) and adjusted the figure legends accordingly (line 743-748). Finally, we have performed the proposed experiment at higher serum concentrations and attached viability and IM-permeabilisation data over time to our manuscript (S2a-c) and revised the text (line 153-155) and figure legends accordingly (line 750-755). We chose an intermediate serum-resistant strain, Kp209, and incubated it with 10% or 20% NHS with and without supplementary C9. We observed >1-log increase in bacterial killing for both serum concentrations upon addition of supplementary C9 after 90 minutes (S2a) and faster IM-permeabilisation of both serum concentrations compared to 5% NHS (S1c, S2b-c). This data confirms our observations of supplementing C9 to NHS are also relevant at higher serum concentrations and over multiple shorter timepoints.

### Reviewer #2:

1. Figure and legends need to better clarify the number of samples used; this is not always evident, and in some figures, this is not sufficient. For example, in Figure 1b: it seems that some data points were performing with only an n=2, which is not sufficient. The experiments need to be repeated with an n=3 at the very minimum.

We thank the reviewer for pointing this out. Some individual experiment dots were too small to be seen in Figure 1b. We have reformatted the individual experiment dots so they are clearly visible. Additionally, the figure legends state that the data are from three independent experiments.

2. Similarly, in Fig. 1c, it is unclear how many samples were used for each time point. This is true for all figures where the averages were shown.

For the line graphs, plotting every individual dot in addition to having the mean and error bars for the line plots results in a visually poor outcome. All figure legends mention the number of samples used for each concentration and the individual values can be found in the supplementary data.

## Part III – Minor Issues: Editorial and Data Presentation Modifications

### Reviewer #1:

1. Figure 2C - I am confused by this figure - I think this is another way to represent their findings in 2A. But if they are representing “killing” should it not be the initial inoculum - final yield for each strain and then measure that ratio for each strain? Rather than just calculating ratio of final yield?  If that is what the authors did, it was not immediately clear. Also, would a ratio <1 indicate impaired killing? That would be how I interpret a ratio, which is not consistent with 2A.

We thank the reviewer for pointing out the unclarity on the interpretation of Fig. 2c. Fig. 2c is intended the represent the relative reduction in bacterial viability additional C9 has per strain, which is reflected by the fold change in CFU/ml with and without C9 in serum. In this experimental set-up, the initial inoculum is consistent for each strain. For example, for KpO1_1, we observe a >10-fold reduction in bacterial viability between the serum and serum+ C9 condition in Figure 2a, which is plotted as >10-fold reduction in bacterial viability by supplementary C9 in Figure 2c.

To clarify, we have adjusted the axis labels and better explain that the value that is displayed was calculated by taking the fold difference between the CFU/ml values of the serum condition and the serum+C9 from Figure 2a and included this in the manuscript text (line 148-149) and figure legend (line 188). We have also added horizontal lines to indicate what constitutes no reduction or 10-fold reduction in bacterial viability by supplementary C9 in Figure 2c.

2. Minor point: I am not particularly concerned with statistical significance when the authors are showing multi-log differences in bacterial killing but in Figure 2a there seems to be minimal changes that influence their results. Would a non-parametric test like Mann-Whitney change statistical significance? This is a minor point.

We agree that non‑parametric tests are appropriate when data do not meet assumptions of normality. However, in our experimental design each data point represents a paired biological measurement (the same bacterial replicate assayed under 5% NHS and NHS + C9). Because of this, the correct non‑parametric alternative would be the Wilcoxon matched‑pairs signed‑rank test, rather than the Mann–Whitney U test. However, since our sample size per strain is n = 3–4 paired biological replicates. the Wilcoxon test is statistically valid but has extremely coarse exact p‑values (the smallest possible two‑sided p is 0.125 for n = 4, and 0.25 for n = 3). For this reason, Wilcoxon lacks power in this context, even when all replicates show a consistent directional effect.

The reviewer also notes that “minimal changes” appear significant. This reflects the fact that the paired design captures consistent within‑replicate shifts, even if the absolute magnitude of change is modest. When analyzed appropriately as paired data, these small but reproducible shifts yield statistical significance.

For these reasons, we maintain the use of the paired t‑test for the per‑strain comparisons. Importantly, as the reviewer mentioned, this choice does not affect the biological interpretation, the multi‑log reductions in killing observed for several strains are reproducible and remain evident regardless of statistical framework.

3. Lines 466-468: Regarding neonatal supplementation of C9, would recommend testing in pre-clinical animal models to assess its effect in vivo.

We have revised the discussion text to include this suggestion (line 435-436)

4. For future submissions, It would be helpful to have figure legends co-located with the figures. Or at the end of the text rather than embedded in the text without the adjacent figure.

The PLOS pathogens submission guidelines state that we upload the figures as individual tiff files and place the figure legends under the results section per figure, therefore we did not embed the figure legends with the figures.

Reviewer #2: 2. The strains in figure 2 belong to many O and K antigens. Given that capsules are important for complement resistance, can the authors include any information on the K antigens of the strains? Are some of the strains hypermucoviscous? Some appear to be quite resistant to killing.

Yes, K type is mentioned in Table 1 and Table S1. We performed the string test to assess if the strains are hypermucoviscous by protocol described in materials & methods (line 460-464). None of the strains tested positive for hypermucoviscosity (HMV) using the string test (Table S1). Additionally, the genotyping data shows that all strains are absent of the HMV genes further demonstrating that these strains are not hypermucoviscous (Table S1).

3. In Fig. 4D, it seems that killing is not restored to baseline levels without C9 even when IgG1 UKpn2 is above 0.1ug/ml. Please clarify and/or revise the data interpretation

The reviewer is correct to point out that the correct statement in line should be above 0.3 µg/ml, which we have adjusted accordingly to clarify the interpretation of these data (line 280-281).

We now performed a one-way ANOVA’s with Tukey’s multiple comparisons test to compare all 3% dNHS conditions without supplementary C9 to the 0-antibody condition which revealed no significant difference (p=0.99) between 1 µg/ml of UKpn2 and 0 µg/ml in serum without C9, confirming a return to baseline level. This statistical test has now been added to the figure and figure legend for clarity (Fig. 4d; line 302-304).
